# Temporary Hemiplegia

**Published:** 1840-10

**Authors:** 


### TEMPORARY HEMIPLEGIA AND APHONIA, APPARENTLY FROM WORMS.

Dr. A. A. Patterson, of Franklin county Kentucky, in a letter to Dr. Drake, communicates the following case:

"September 6th, 1838, I was sent for in haste, by Mr. D. to visit his son, ten years old, whom I found totally paralytic in the right side and unable to speak. Desiring to see his tongue, he was unable to protrude it, and I had actually to bring it forward with my fingers—it was coated with a white fur to its edges, which were preternaturally red. His pulse beat 115 strokes in a minute. The expression of his countenance was rather idiotic. During the day, before my arrival, he had vomited four or five lumbricoides.

"I immediately ordered an emetic, which caused the discharge of several additional worms, with some bile. I then administered a dose of calomel and jalap, eight grains each; and left two additional portions of the former medicine, each nine grains, to be exhibited at intervals of two hours.

"When I called the next day, my patient was able to walk about and speak. I prescribed a dose of castor oil; and when I saw him a few days afterwards, he was quite well." D.

